# Clinical and Molecular Comparative Study of Colorectal Cancer Based on Age-of-Onset and Tumor Location: Two Main Criteria for Subclassifying Colorectal Cancer

**DOI:** 10.3390/ijms20040968

**Published:** 2019-02-22

**Authors:** Edurne Álvaro, Juana M. Cano, Juan L. García, Lorena Brandáriz, Susana Olmedillas-López, María Arriba, Daniel Rueda, Yolanda Rodríguez, Ángel Cañete, Julia Arribas, Lucía Inglada-Pérez, Jessica Pérez, Carlos Gómez, Mariano García-Arranz, Damián García-Olmo, Ajay Goel, Miguel Urioste, Rogelio González-Sarmiento, José Perea

**Affiliations:** 1 Surgery Department, “Infanta Leonor” University Hospital, 28031 Madrid, Spain; eac.lomba@gmail.com; 2 Oncology Department, Ciudad Real General Hospital, 13005 Ciudad Real, Spain; juanamariacano@gmail.com; 3 Molecular Medicine Unit- Department of Medicine, Biomedical Research Institute of Salamanca (IBSAL), University of Salamanca-SACYL-CSIC, 37007 Salamanca, Spain; jlgarcia@usal.es (J.L.G.); jperezg84@hotmail.es (J.P.); 4 Surgery Department, “Fundación Jiménez Díaz” University Hospital, 28040 Madrid, Spain; lorena.brandariz@gmail.com; 5 Health Research Institute-Fundación Jiménez Díaz University Hospital, 28040 Madrid, Spain; susana.olmedillas@fjd.es (S.O.-L.); mariano.garcia@quironsalud.es (M.G.-A.); 6 Biochemistry Department, Gregorio Marañon University Hospital, 28007 Madrid, Spain; arribadomenech@gmail.com; 7 Molecular Biology Laboratory, University Hospital “12 de Octubre”, 28041 Madrid, Spain; druedafer.hdoc@gmail.com; 8 Digestive Cancer Group, “12 de Octubre” Research Institute, 28041 Madrid, Spain; 9 Pathology Department, University Hospital “12 de Octubre”, 28041 Madrid, Spain; yolandarodriguezgil@hotmail.com; 10 Gastroenterology Department, University Hospital “12 de Octubre”, 28041 Madrid, Spain; canete_angel@hotmail.com (Á.C.); jantiart@gmail.com (J.A.); 11 Centre for Biomedical Network Research on Rare Diseases (CIBERER), Institute of Health Carlos III, 28029 Madrid, Spain; linglada@cnio.es; 12 Oncology Department, University Hospital “12 de Octubre”, 28041 Madrid, Spain; cgomezm@seom.org; 13 Center for Gastrointestinal Research, Center for Translational Genomics and Oncology, Baylor Scott & White Research Institute, Charles A. Sammons Cancer Center, Baylor University Medical Center, Dallas, TX 75246, USA; 14 Familial Cancer Clinical Unit, Spanish National Cancer Centre (CNIO), 28029 Madrid, Spain

**Keywords:** early-onset colorectal cancer, late-onset colorectal cancer, microsatellite instability, chromosomal instability, CpG island methylator phenotype, tumor location.

## Abstract

Our aim was to characterize and validate that the location and age of onset of the tumor are both important criteria to classify colorectal cancer (CRC). We analyzed clinical and molecular characteristics of early-onset CRC (EOCRC) and late-onset CRC (LOCRC), and we compared each tumor location between both ages-of-onset. In right-sided colon tumors, early-onset cases showed extensive Lynch syndrome (LS) features, with a relatively low frequency of chromosomal instability (CIN), but a high CpG island methylation phenotype. Nevertheless, late-onset cases showed predominantly sporadic features and microsatellite instability cases due to *BRAF* mutations. In left colon cancers, the most reliable clinical features were the tendency to develop polyps as well as multiple primary CRC associated with the late-onset subset. Apart from the higher degree of CIN in left-sided early-onset cancers, differential copy number alterations were also observed. Differences among rectal cancers showed that early-onset rectal cancers were diagnosed at later stages, had less association with polyps, and more than half of them were associated with a familial LS component. Stratifying CRC according to both location and age-of-onset criteria is meaningful, not only because it correlates the resulting categories with certain molecular bases, but with the confirmation across larger studies, new therapeutical algorithms could be defined according to this subclassification.

## 1. Introduction

In Europe, colorectal cancer (CRC) is the third most common cancer in males, the second most common cancer in females, and the fourth most common cause of cancer-related deaths; worldwide, 1,360,000 new cases are diagnosed each year and it causes 700,000 deaths [1]. Biologically, it represents a heterogeneous disease. Early-onset colorectal cancer (EOCRC) represents 11% of colon cancers and 18% of rectal cancers, and its incidence is increasing [2]. Several studies have described different genetics, biological and clinical behavior in this age group, suggesting that it may be a specific subgroup within CRC, and that age-of-onset should be a major criterion for its subclassification [3,4]. Moreover, new findings may also define some particular subtypes, such as rectal cancer with microsatellite-stability (MSS) and without chromosomal instability (CIN-) [5]. 

The predisposition of the three main carcinogenetic CRC pathways to different locations in the colon, together with studies demonstrating that right and left-sided CRCs exhibit different genetic, biological and demographical characteristics and risk factors, suggest that the carcinogenetic mechanism and progression of CRC may differ with tumor location. Thus, the anatomic site of origin of CRC appears to be another good discriminator for the subclassification of this type of neoplasm [6,7,8]. Along these lines, we recently analyzed tumor location (right colon, left colon and rectum) as a discriminatory factor within EOCRC and clinical differences emerged as well as the different main carcinogenetic pathways predominant within each location [9].

For the past few years, while characterizing EOCRC from different points of view, we have been comparing series of features with their correlates in late-onset CRC (LOCRC) in order to assess whether they have a different molecular basis [3,4,10]. Therefore, our purpose at this point is to confirm whether our previous findings that the differential clinical and molecular features of tumors with a different location in CRC and the age-of-onset as a major criterion to classify CRC, hold up together. If so, right colon, left colon and rectal cancers should be substantially different between early and late age-of-onset groups.

## 2. Results

### 2.1. EOCRC: Global Group Features and Comparative Analysis According to Colon Location

#### 2.1.1. Clinicopathological and Familial Features

Comparative analysis of EOCRC with respect to tumor location has been published before [9]. Left colon was the most frequent location (43%), followed by rectum (33%). The main features for each location are shown in Table 1, Table 2 and Table 3. 

#### 2.1.2. Molecular Features

Approximately 15% of EOCRC tumors showed Microsatellite Instability (MSI); the most prevalent location was the right colon (30%), followed by the left colon (17%). None of the tumors located at the rectum showed MSI. Most MSI cases were due to LS (germline mutations in MMR genes). CIMP-high was also located mainly in the right colon (50%). Therefore, according to the molecular classification, the most homogeneous distribution was observed for right colon cancers. For left colon and rectal cancers, the largest category was MSS-CIMP-Low/0 (77.5% and 90.5%, respectively). Right colon tumors showed the lower CIN, whereas left colon tumors exhibited the highest CIN, particularly with respect to losses. Rectal tumors showed the highest mean number of whole chromosome aberrations. 

As we published before, recurrent gains and losses were more frequent in right-sided early-onset colon cancers (EOCCs) [9]. The only recurrent region that was observed in all three tumor locations was the gain of 19p13.3-q13.24. Five regions were common to right- and left-sided EOCCs: losses at 1q12-q21.2, 5q13.2, 9p12-p13, 9p13.1, and 10q11.22. Other frequently altered regions were: gains at 7q22.1 and losses at 16p13.12-p12.3 in right-sided EOCCs; losses at 9q21.11 and 11q14.2-14.3, and gains at 20q11.21-q11.22 in left-sided EOCCs; losses at 14q11.1-11.2, and gains at 17q21.31-q21.32 and 22q11.1 in early-onset rectal cancers (EORCs) [9]. 

### 2.2. LOCRC: Global Group Features and Comparative Analysis According to Colon Location 

#### 2.2.1. Clinico-Pathological and Familial Features

Clinical and molecular features of global LOCRC and comparison between colon locations have been published before [4,9,10,11], and are summarized in Table 1, Table 2 and Table 3. The least frequent location was left colon (23%), whereas the other two locations were observed at equivalent rates.

#### 2.2.2. Molecular Features

With regard to the three main molecular carcinogenetic pathways, nine cases showed MSI (9.3%), with only one of them due to an MMR-germline mutation; seven of the others were due to *BRAF* mutations and/or hypermethylation of the *MLH1* promoter, the majority of which were located in the right colon. With regard to the molecular classification, the highest proportion was related to MSS-CIMP-Low-0, reaching almost 86% of all left colon cancers. Interestingly, another frequently occurring molecular classification was MSS-CIMP-High in rectal tumors, reaching 36%. The highest CIN was observed in the right colon. Left colon cancers showed an interestingly low CIN, and rectal tumors showed a high mean of whole altered chromosomes.

Recurrent gains and losses were slightly more frequent in right-sided late-onset colon cancers (LOCCs), followed by rectal cases. The only recurrent regions that were observed in all three tumor locations were gains of 7q11.22-11.23 and 20q11.1-q11.23. Four regions were common to right- and left-sided LOCCs: losses at 18q12.1-12.2, 18q21.1 and 18q22.1-23, and gains at 7p22.1. Only two regions were common to left-sided LOCCs and late-onset rectal cancers (LORCs): losses at 5p15.33-15.31 and alterations in 1q21.1-21.2. Finally, a high proportion of altered regions were common between the right colon and rectal cancers. Individually, the most frequently altered regions for each location were gains at 7q11.21 and 7q11.23, and losses at 18q22.1-22.3 in right-sided LOCCs; losses at 10q11.21-11.23 in left-sided LOCCs; gains at 7q11.22-11.23 in LORCs. 

### 2.3. Right Colon Cancers: Comparison between EOCC and LOCC

#### 2.3.1. Clinico-Pathological and Familial Features

We compared right colon cancers between both age-of-onset populations (Table 1). The right colon location was more frequent in LOCRC (39% vs 24%). Most of the differential features related to the early-onset subset were because of the LS component: cases were diagnosed at early stages, with a higher mean number of polyps during follow-up and showed an important familial component. They also showed a better disease-free survival (DFS). On the other hand, late-onset cases developed on average fewer polyps during follow-up, and three-quarters of them were sporadic.

#### 2.3.2. Molecular Features

**Figure 1:**
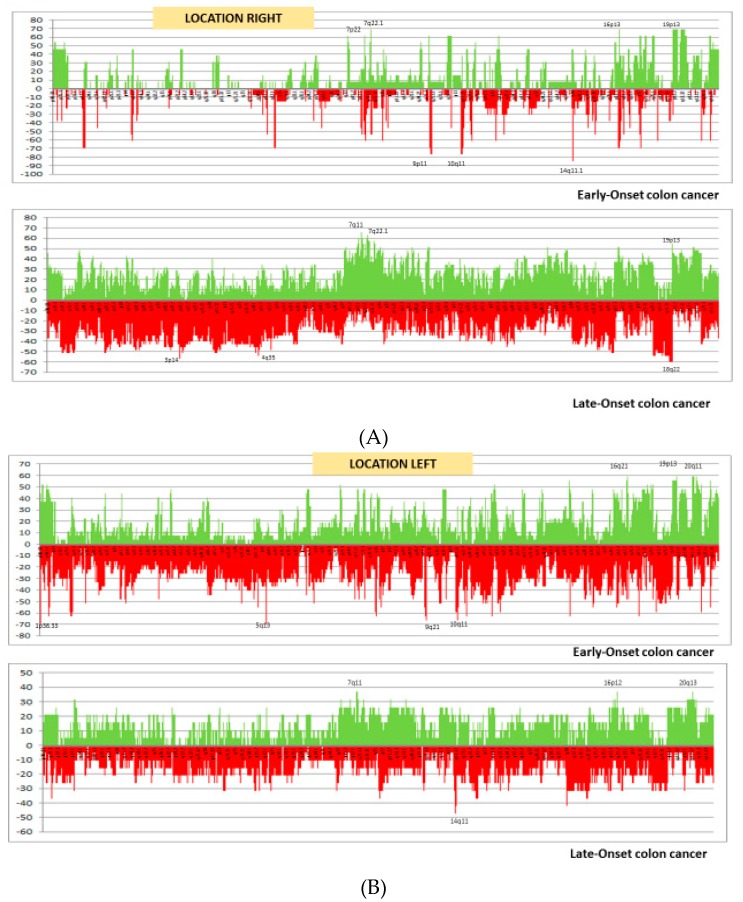

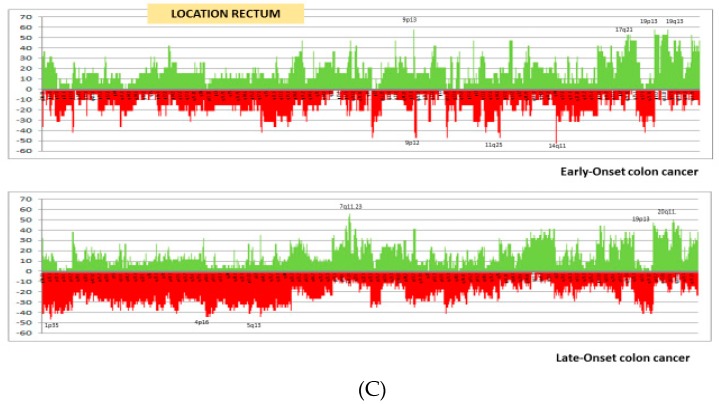
Frequency plots of copy number gains (above zero, green) and losses (below zero, red) defined for each subgroup. The fraction gained or lost is plotted on the *y*-axis versus genomic location on the *x*-axis. (**A**) Comparison of right-sided cancers in EOCC and LOCC; (**B**) comparison of left-sided cancers in EOCC and LOCC; (**C**) comparison of rectal cancers in EORC and LORC. EOCC: Early-onset colon cancer; EORC: Early-onset rectal cancer; LOCC: Late-onset colon cancer; LORC: Late-onset rectal cancer.

With respect to the molecular component of right-sided EOCC, all MSI tumors were due to germline mutations in the MMR genes (30%), with the genomic instability index (GII) being equivalent to a low CIN. Of note, there is a considerable component of CIMP-High in this particular group. All of these features are shown in Table 1. Within right-sided LOCC, the MSI component (18%) was almost entirely due to *BRAF* mutations and/or hypermethylation of the *MLH1* promoter; these cases display some already known particular characteristics, such as a CIMP-High component [12]. This subset showed a much higher CIN compared with its EOCC equivalent, as shown in Figure 1A.

The right colon is the location with most differentially altered chromosomal segments according to the age of onset, mainly for the late-onset group (Table S1), and the most frequent specific differential segments for each location are shown in Table 4. From a total of 113 differentially altered chromosomal segments, 100 corresponded to right-sided LOCC, and losses in chr2 and gains in chr7 were the most frequent. Only 13 differential segments were particularly predominant in right-sided EOCC, in most cases corresponding to losses in 1q21.1-21.2, 10q11.21-11.22 and 14q11.1-11.2.

### 2.4. Left Colon Cancers: Comparison between EOCC and LOCC

#### 2.4.1. Clinico-Pathological and Familial Features

The age subset with the highest proportion of left colon cancers was EOCRC (43%). Most of the clinicopathological and familial features were similar for both groups, as shown in Table 2. The earlier stages at diagnosis in the left-sided EOCC group (80% for stages I and II), and the higher development of polyps as well as other CRCs (S and/or MCRC) in left-sided LOCC were the differential characteristics. 

#### 2.4.2. Molecular Features

The higher CIN in the early-onset subgroup is noteworthy (Figure 1B and Table 2). A very low number of differentially altered chromosomal segments between the age-of-onset groups was observed, mainly in the early-onset subset. Differential segments are listed in Table S1 and summarized in Table 4. In early-onset subgroup, losses were present in most cases: in 1p12-q21.1, 5q13.1-13.2, 9p12-q24.22, 9q31.3-33.1, 11p11.12-q12.1 and 11q14.41-14.3, as well as a gain in 19p13.12-12.

### 2.5. Rectal Cancers: Comparison between EORC and LORC

#### 2.5.1. Clinicopathological and Familial Features

Table 3 summarizes the main differential features between rectal cancers of both age-groups. From a clinical point of view, EORCs showed less association with polyps, a very advanced stage at diagnosis (37% with metastasis), and a better DFS; a relevant aspect was the important familial cancer component, with more than 50% of cases with LS-related neoplasms in their families. Conversely, LORC cases were mainly sporadic. 

#### 2.5.2. Molecular Features

All LORCs were MSS, and an important number of these tumors showed CIMP-High (36%). More than 90% of EORC were MSS-CIMP0. Lastly, there were differences in relation to CIN, with the young rectal cancers displaying less instability for losses (Figure 1C and Table 3).

Although there were some differentially altered chromosomal segments between these age-of-onset groups, they were not as high in proportion as in other locations, with a slight predominance for LORC (Table 4 and Table S1). In this last group losses in 1p36.32-36.13 and gains in 5q13.2 are the most frequent. 

## 3. Discussion

CRC is a heterogeneous disease with different outcomes and drug responses. Subclassification according to the age of onset and tumor location may have applications in terms of diagnosis, prevention and therapy. We recently published differential features for EOCRC and LOCRC separately, according to tumor location, and some interesting subsets within these age-of-onset groups became manifest [9,11]. More interesting, however, was the possibility that both factors, age of onset and colon location may differentiate CRC.

In right colon cancers, the differential phenotypes for both age groups are clearly correlated with their molecular basis. Right-sided early-onset cases showed important LS features (poorly differentiated tumors, a higher mean number of polyps, the familial component of LS-related neoplasms), all of which are likely to be associated with the 30% of MSI due to germline mutations in MMR genes; these tumors have a relatively low CIN but are CIMP-high. The late-onset subset was mainly present in females, with sporadic features, and only 18% of cases were MSI; this was seldom due to LS but mostly because of *BRAF* mutations. In spite of the higher CIN of right-sided LOCCs, the two most frequently occurring differential regions (1q21 and 10q11.21-11.22) were observed in early-onset cases. Increased alterations in 1q21 have been found before in EOCRC, although the criterion of tumor location was not applied [13], while 10q11.21-11.22 is a region where the *RET* gene is located [14]. Another important aspect that should be underlined is the presence of losses in 14q11.1-11.2. Here the *NDRG2* gene is located, a tumor suppressor gene that has been associated with *c-MYC* and the TGF-β pathway in colorectal carcinogenesis [15,16], and its epigenetic silencing promotes tumor proliferation and invasiveness [17].

Left-colon tumors showed predominantly differences according to CIN (mainly whole chromosomes altered). The most reliable clinical features were associated with the late-onset subgroup, showing an important tendency to develop polyps as well as Multiple Primary CRC, in contrast with the early-onset subgroup. Nevertheless, left-sided EOCCs showed the higher CIN, as well as some differential CNAs, few of which have been associated previously with other features of CRC or the early-onset of cancers. As we mentioned before, losses in 1q21.1-21.2 are found significantly more in EOCRC [13]; losses in 11q14.1-14.3 have been associated with aggressive behaviors and familial linkage, albeit in prostate cancers [18]; gains in 19p13.12 may be related with *NOTCH3* expression or function [19]. In addition, 11q14.1 harbors *GAB2*, a gene involved in Epithelial to Mesenchymal transition, and more importantly, in the development of lymph-nodes invasion and metastasis [20].

Differences within rectal cancers are more prominent in the early-onset subset: later-stages at diagnosis, less association with polyps, and more than half of them associated with a familial LS component, although all of them are MSS. Only the CIMP-High-MSS category (36%) within LORC is noteworthy. Only a few differential chromosomal segments appeared at high rates, and the most frequent were associated with LORC; one of these, gains in 5q13.2, involves a segment associated with rectal metastatic carcinomas [21].

Finally, we wanted to assess to which categories our location and age-of-onset subgroups corresponded when comparing them with the recent consensus molecular classification of the CRC subtyping consortium [22]. Since consensus molecular subtype 1 (CMS1 or MSI immune) is mainly located in the right colon, we compared both age groups. As CMS1 characterized by MSI, this is clearly related to both age groups, but mainly with the right-sided early-onset subgroup. The other features are shared by the separate groups individually. While right-sided EOCC is linked with CIMP-High and a low prevalence of somatic CNAs or CIN, right-sided LOCC showed *BRAF* mutation, high grade of differentiation, predominance in females, and worse survival after relapse, in agreement with recent studies showing worse prognosis of patients with MSI and recurring *BRAF*-mutant CRC [23,24,25]. In our study, the location group within EOCRC that had the best prognosis was the right colon, while for LOCRC it was the left colon.

Consensus molecular subtype 2 (CMS2 or Canonical) is the most frequent and is defined by somatic copy number alterations (SCNAs) in a larger proportion than in the other subtypes, by being mainly left-sided and by having a larger proportion of long-term survivors. Compared to our study, the first three characteristics are clearly connected with left-sided EOCC and only the last with left-sided LOCC.

Consensus molecular subtype 3 or metabolic type is characterized by MSI, CIMP-Low and low CNA. Some features are associated with EOCRC (left colon with CIMP-low and MSI, and rectum also CIMP-low), and others with left-sided LOCC and LORC, respectively (low CNA and CIMP-High).

Consensus molecular subtype 4 or mesenchymal type exhibits SCNA high, is diagnosed at more advanced stages and show worse relapse-free and overall survival. Rectal tumors mainly with early-onset could be classified in this molecular subtype. This point about rectal tumors deserves a separate consideration to the extent that CMS classification mainly focused on right versus left colon cancer, and only a few rectal cancers were included. The study of rectal cancer within EOCRC may arise especially significant, as this subtype is the one that is increasing more remarkably in the last years [26,27]. Other important points to take into account are that it has the worse prognosis for both age-of-onset subgroups and that rectal cancer is a different disease than colon cancer. [28]. Efforts are needed in order to identify with enough cases the group of the aforementioned classification where rectal cancer could be framed if a new category does not arise as a result of this analysis.

However, our results and their correlative implications should be considered cautiously given the limited sample size of the groups. Additional studies with a larger sample size should be developed to confirm our findings.

## 4. Materials and Methods 

### 4.1. Families, Samples and Data Collection

Between January 2002 and December 2008, a total of 82 consecutive individuals with CRC diagnosed at an age of 45 years or younger (EOCRC) (we excluded 6 cases diagnosed with familial adenomatous polyposis), and 97 consecutive patients with CRC diagnosed at an age of 70 years or older (LOCRC), were collected at our institution. They were considered the index case of each family. 

The age of 45 years or younger was chosen as an inclusion criterion because although there is no consensus on a specific age, according to the literature, it is the age range where the majority of genetic and phenotypic alterations are found. The age of 70 years or older was chosen as an inclusion criterion because according to the World Health Organization, 65–70 years old is the cut-off point from which a patient is considered older. Patients between 45 and 70 years of age were not included since, according to the literature, cases may behave like late genetic syndromes or attenuated or more frequently as sporadic tumors, thus overlapping with both groups. This may be a cause of confusion factor that leads to false conclusions [29,30,31,32]. 

All subjects gave their informed consent for inclusion before they participated in the study. The study was conducted in accordance with the Declaration of Helsinki, and the protocol was approved by the Ethics Committee of the “12 de Octubre” University Hospital. Personal and clinicopathological information was obtained including age of onset, gender, location of the CRC (right/left colon or rectum), grade of cell differentiation (low, medium or high), mucin production, the presence of “signet ring” cells, TNM stages (6th version), the existence of polyps during follow-up, type of polyps (adenomatous, hyperplastic or mixed), the presence of synchronous or metachronous CRCs (SCRC or MCRC), and primary multiple neoplasms in the index case. The tumors (adenocarcinomas) were pathologically confirmed by a single pathologist.

Right colon tumors have been defined as those ranging from the caecum to the proximal two-thirds of the transverse colon and left colon tumors as those ranging from the distal third of the transverse colon, including the splenic flexure, to the sigmoid colon. Rectal cancers are defined as those until 16 cm from anal verge (rectosigmoid junction).

To analyze the antecedents of cancer, families were classified into four groups: (a) families fulfilling the Amsterdam II criteria for Lynch syndrome (LS) [33]; (b) families with mainly aggregation—at least one in first-degree or two in second-degree family members of LS-related neoplasms; (c) families with mainly aggregation of LS-unrelated neoplasms; (d) cases without oncological antecedents; these were considered sporadic cases.

Follow-up was at least 5 years from surgery; DFS and overall survival (OS), recurrence and cancer-related death were recorded for each case and were included in the different attached tables as a mean.

### 4.2. Microsatellite Instability and Mutational Analysis

A microsatellite instability (MSI) analysis was performed using the Bethesda panel [34], as published before [3]. Tumors were considered as MSI when showing high-frequency MSI (MSI-H; two or more of the five markers showing instability), while the rest (including MSI-L) were classified as MSS.

MSI cases were screened for germline mutations in the mismatch repair (MMR) system genes *MLH1*, *MSH2* and *MSH6* as previously reported, with minor modifications [35].

Sporadic MSI cases were identified by determining the methylation status of the *MLH1* gene promoter, as well as assessing the V600E mutation in the *BRAF* gene. The methods used were described previously [4].

### 4.3. Analysis of CpG Island Methylation Phenotype Panel

Methylation status of promoter regions of the CpG island methylation phenotype (CIMP) panel genes *CACNA1G*, *CDKN2A*, *CRABP1*, *IGF2*, *MLH1*, *NEUROG1*, *RUNX3* and *SOCS1* were analyzed as published before [4]. CIMP-High was defined as the presence of ≥6/8 methylated promoters, CIMP-Low as 1/8 to 5/8 methylated promoters, and CIMP-0 as the absence (0/8) of methylated promoters.

### 4.4. Molecular Classification

Based on the MSI and CIMP status, we divided both age groups into four categories because differences between MSI-L and MSS are subtle and differences between CIMP-Low and CIMP-0 are also subtle: (MSI/CIMP-High); (MSI/CIMP-Low/0); (MSS/CIMP-High); and (MSS/CIMP-Low/0) [12].

### 4.5. Chromosomal Instability. Array Comparative Genomic Hybridization (aCGH)

A CGH was performed using oligonucleotide microarrays (Roche NimbleGen, Inc., Reykjavik, Iceland) in order to identify copy number alterations (CNA) for both age-of-onset subgroups and has been described before [10]. The degrees of genomic instability were also described in that same study. Both datasets were included in the gene expression omnibus (GEO): LOCRC (GSE108166) and EOCRC (GSE108220).

### 4.6. Statistical Analyses

Continuous variables were expressed as mean values plus/minus standard deviation (SD), and categorical variables were expressed as the number of cases and their percentage. Differences were considered significant when *p* < 0.05. For associations between colon location and other discrete variables, statistical analyses were performed using Pearson’s chi-square (χ^2^) test for parametric variables, and Fisher’s exact test for non-parametric variables. When those features were continuous variables, Student’s *t*-test was used. The SPSS v.11.5 for Windows (SPSS, Inc., Chicago, IL, USA) statistical package was used.

For the CGH analysis, both univariate and multivariate analysis was carried out to identify significant minimum regions. Regarding the univariate analysis, unconditional logistic regression was done for each candidate region. For the multivariate analysis, each of the regions was tested separately, including other relevant clinical variables. Location was considered a factor in all analyses carried out. We selected only regions larger than 1 Mb, to avoid any possible bias. This analysis was performed in R Statistical Software [36].

## 5. Conclusions

In our opinion, dividing CRC according to both location and age-of-onset criteria is meaningful, not only because it homogenizes the resulting categories, but also because it facilitates new approaches in those subsets of which the molecular basis remains as yet unknown: high CIN left-sided EOCC, left-sided LOCC with multiple primary neoplasms, or EORC with a familial component and advanced stage at diagnosis. The subclassification also helps to define clinically which tumors are more likely to show certain already described molecular alterations, such as for example changes affecting *NDRG2* or *GAB2*. Comparison with the consensus molecular classification also confirms the importance of considering both criteria when studying CRC. With the confirmation across larger studies, new therapeutical algorithms could be defined according to the classification of the age-of-onset and the location of the tumor.

## Figures and Tables

**Table 1:** Comparison between right colon cancers with different ages of onset.

RIGHT COLON	n/N EOCRC	EARLY-ONSET n/Right-sided EOCC (%)	n/N LOCRC	LATE-ONSET n/ Right-sided LOCC (%)	p(χ^2^)
**Number of cases (%)**	20/82	24%	38/97	39%	
**Age of Onset (mean (SD), years) ^1^**	-	39.1 (6)	-	79.08 (5)	NS
**Gender**	10/82	Female 10/20 (50%)	25/97	Female 25/38 (66%)	NS
**Grade of tumor differentiation (%) ^2^**	5/82	Poor 5/20 (25%)	24/97	Medium 24/38 (63%)	NS
**Mucosecretion^2^**	7/82	7/20 (35%)	13/97	13/38 (34%)	NS
**Presence of “Signet-ring” cells ^2^**	0/82	0/20 (0%)	0/97	0/38 (0%)	NS
**TNM (UICC) Stage (%)**	16/82	I-II: 16/20 (80%)	30/97	II-III: 30/38 (79%)	NS
**Associated Polyps**	7/82	14/20 (70%)	19/97	24/38 (63%)	NS
**Mean number of associated polyps ^1^**	-	**3**	-	**1.8**	**0.021**
**Type of associated polyps (%)**	10/82	Mixed: 10/20 (50%)	21/97	Adenomatous: 21/38 (54%)	NS
**S- and/or MCRC**	4/82	4/20 (20%)	9/97	9/38 (24%)	NS
**Amsterdam criteria for LS**	8/82	**8/20 (40%)**	1/97	**1/38 (3%)**	**<0.001**
**Familial aggregation for LS-related neoplasms**	7/82	**7/20 (35%)**	6/97	**6/38 (16%)**	**0.008**
**Familial aggregation for LS-unrelated neoplasms**	1/82	1/20 (5%)	1/97	1/38 (3%)	NS
**Sporadic cases**	4/82	**4/20 (20%)**	28/97	**28/38 (74%)**	**<0.001**
**Disease-Free Survival (mean; months) ^1^**	-	**72.4**	-	**20.16**	**0.002**
**Global Survival (mean; months) ^1^**	-	**81.06**	-	**45.94**	**0.003**
**MSI**	6/82	6/20 (30%)	7/38	7/38 (18%)	NS
**MMR genes germline mutations**	6/82	6/20 (30%)	5/97	5/ 38 (13%)	NS
***BRAF* mutation**	0/82	0/82 (0%)	5/97	5/38 (13%)	NS
**CIMP Classification (%) ^2^**					**0.025**
**High**	**8/78**	**8/16 (50%)**	7/90	7/36 (20%)	
**Low**	6/78	6/16 (37.5%)	12/90	12/36 (33%)	
**0**	2/78	2/16 (12.5%)	**17/90**	**17/36 (47%)**	
**Molecular Classification (%) ^2^**					
MSI-CIMP-High	3/82	3/16 (19%)	5/90	5/36 (14%)	**0.019**
MSI-CIMP0	2/82	2/16 (12.5%)	2/90	2/36 (5%)	
**MSS-CIMP-High**	**5/82**	**5/16 (31%)**	2/90	2/36 (5%)	
**MSS-CIMP0**	**6/82**	**6/16 (37.5%)**	**27/90**	**27/36 (75%)**	
**Genomic instability^1^**					
**GII Gain**		0.144296		0.183649	NS
**GII Loss**		0.058762		**0.249820**	**0.003**
**GII Normal**		**0.796933**		0.566522	**0.03**
**Mean Gained Chromosomes**		1.38		1.80	**NS**
**Mean Lost Chromosomes **		0.08		**2.20**	**0.18**

^1^ Statistical analysis was carried out using Student’s *t*-test. ^2^ It has been carried out on the maximum number of patients with the specimen available. EOCC: Early-onset colon cancer; EOCRC: Early-onset colorectal cancer; LOCC: Late-onset colon cancer: LOCRC: Late-onset colorectal cancer; NS: Not significant; SD: Standard deviation; UICC: International Union Against Cancer; S- and/or MCRC: synchronous and/or metachronous; LS: Lynch syndrome; MMR: Mismatch repair; MSI: Microsatellite instability; CIMP: CpG island methylator phenotype; MSS: Microsatellite stable; GII: Genomic instability index.

**Table 2:** Comparison between left colon cancers with different ages of onset.

LEFT COLON	n/N EOCRC	EARLY-ONSET n/Left colon EOCC (%)	n/N LOCR	LATE-ONSET n/ Left colon EOCC (%)	p(χ^2^)
**Number of cases (%)**	35/82	43%	22/97	22.7%	
**Age of Onset (mean (SD), years) ^1^**	-	39.3 (4.2)	-	74.86 (5)	NS
**Gender (%)**	22/82	Male 22/35 (63%)	14/97	Male 14/22 (64%)	NS
**Grade of tumor differentiation (%) ^2^**	24/82	Medium 24/35 (68.5%)	17/97	Medium 17/22 (77%)	NS
**Mucosecretion ^2^**	8/82	8/24 (33%)	2/97	2/22 (9.1%)	NS
**Presence of “Signet-ring” cells ^2^**	2/82	2/24 (8%)	0/97	0/22 (0%)	NS
**TNM Stage (%)**	27/82	**I/II 27/35 (77%)**	18/97	**II-III 18/22 (82%)**	**0.003**
**Associated polyps**	21/82	21/35 (60%)	13/97	13/22 (59.1%)	NS
**Mean number of associated polyps ^1^**	-	**1.6**	-	**5.41**	**<0.001**
**Type of associated polyps (%)**	10/82	Adenomatous 10/21 (48%)	7/97	Adenomatous 7/13 (54%)	NS
**S- and/or MCRC**	0/82	**0/35**	8/97	**8/22 (36%)**	**<0.001**
**Amsterdam criteria for LS**	6/82	6/35 (17%)	0/97	0/22 (0%)	NS
**Familial aggregation for LS-related neoplasms**	6/82	6/35 (17%)	6/97	6/22 (27%)	NS
**Familial aggregation for LS-unrelated neoplasms**	6/82	6/35 (17%)	2/97	2/22 (9%)	NS
**Sporadic cases**	17/82	17/35 (48%)	12/97	12/22 (55%)	NS
**Disease-Free Survival (mean; months) ^1^**	-	48.70	-	31.68	NS
**Global Survival (mean; months) ^1^**	-	76.67	-	96.95	NS
**MSI**	6/82	6/35 (17%)	2/97	2/22 (9%)	NS
**MMR gene germline mutations**	4/82	4/35 (11%)	0/97	0/22 (0%)	NS
**BRAF mutation**	1/82	1/35 (3%)	1/97	1/22 (4.5%)	NS
**CIMP Classification (%) ^2^**					
**High**	3/78	3/31 (10%)	3/96	3/21 (14%)	NS
**Low**	14/78	14/31 (45%)	4/96	4/21 (19%)	
**0**	14/78	14/31 (45%)	14/96	14/21 (67%)	
**Molecular Classification (%) ^2^**					
**MSI-CIMP-High**	1/78	1/31 (3%)	2/96	2/21 (9.5%)	NS
**MSI-CIMP0**	4/78	4/31 (13%)	0/96	0/21 (0%)	
**MSS-CIMP-High**	2/78	2/31 (6.5%)	1/96	1/21 (5%)	
**MSS-CIMP0**	24/78	24/31 (77.5%)	18/96	18/21 (86%)	
**Genomic instability ^1^**					
**GII Gain**		0.1462817		0.101056	NS
**GII Loss**		0.2294307		0.114539	0.06
**GII Normal**		0.6247970		0.784362	NS
**Mean Gained Chromosomes**		**2.00**		**0.78**	**0.03**
**Mean Lost Chromosomes**		**1.93**		**0.61**	**0.03**

^1^ Statistical analysis was carried out using Student’s *t*-test. ^2^ It has been carried out on the maximum number of patients with the specimen available. EOCC: Early-onset colon cancer; EOCRC: Early-onset colorectal cancer; LOCC: Late-onset colon cancer; LOCRC: Late-onset colorectal cancer; NS: Not significant; SD: Standard deviation; UICC: International Union Against Cancer; S- and/or MCRC: synchronous and/or metachronous; LS: Lynch syndrome; MMR: Mismatch repair; MSI: Microsatellite instability; CIMP: CpG island methylator phenotype; MSS: Microsatellite stable; GII: Genomic instability index

**Table 3:** Comparison between rectal cancers with different ages of onset.

RECTUM	n/N EOCRC	EARLY-ONSET n/ EORC (%)	n/N LOCRC	LATE-ONSET n/ LORC (%)	p(χ^2^)
**Number of cases (%)**	27/82	33%	37/97	38.1%	
**Age of Onset (mean (SD), years) ^1^**	-	40.2 (5)	-	78.54 (5)	NS
**Gender (%)**	17/82	Male 17/27 (63%)	23/97	Male 23/37 (62%)	NS
**Grade of tumoral differentiation (%)^2^**	18/82	Medium 18/27 (68%)	29/97	Medium 29/37 (79%)	NS
**Mucosecretion^2^**	5/75	5/20 (25%)	4/97	4/34 (11.8%)	NS
**Presence of Signet-ring cells^2^**	2/75	2/20 (10%)	0/97	0/37 (0%)	NS
**TNM Stage (%)**	10/82	**IV 10/27 (37%)**	18/97	**II 18/37 (49%)**	**0.002**
**Associated Polyps**	11/82	**11/27 (41%)**	25/97	**25/37 (68%)**	**0.04**
**Mean number of associated polyps^1^**	-	1.22	-	2.00	NS
**Type of associated polyps (%)**	7/66	Adenomatous: 7/11 (64%)	18/85	Adenomatous: 18/25 (72%)	NS
**S- and/or MCRC**	0/82	0/27 (0)	4/97	4/37 (11%)	NS
**Amsterdam criteria for LS**	1/82	1/27 (4%)	0/97	0/37 (0)	NS
**Familial aggregation for LS-related neoplasms**	14/82	**14/ 27 (52%)**	0/97	**0/37 (0)**	**<0.001**
**Familial aggregation for LS-unrelated neoplasms**	2/82	2/27 (7%)	3/97	3/37 (8%)	NS
**Sporadic Cases**	10/82	**10/27 (37%)**	34/97	**34/37 (92%)**	**<0.001**
**Disease-Free Survival (mean; months)^1^**	-	**35.85**	-	**16.25**	**<0.001**
**Global Survival (mean; months)^1^**	-	**64.79**	-	**31.67**	**<0.001**
**Microsatellite stability:**	0/82	0/27	0/97	0/37	NS
**MMR gene germline mutat** **ions**	0/82	0/27	0/97	0/27	NS
**BRAF mutation**	0/82	0/27	1/97	1/37 (2.7%)	NS
**CIMP Classification (%)^2^**					NS
**High**	2/76	2/21 (10%)	12/93	12/33 (36%)	
**Low**	8/76	8/21 (38%)	9/93	8/33 (24%)	
**0**	11/76	11/21 (52%)	13/93	13/33 (40%)	
**Molecular Classification (%)^2^**					**0.035**
**MSI-CIMP-High**	0/76	0/21	0/93	0/33	
**MSI-CIMP0**	0/76	0/21	0/93	0/33	
**MSS-CIMP-High**	2/76	2/21 (9%)	12/93	**12/33 (36%)**	
**MSS-CIMP0**	19/76	**19/21 (90.5%)**	21/93	**21/33 (64%)**	
**Genomic instability ^1^**					
**GII Gain**		0.1627359		0.133332	NS
**GII Loss**		0.1441914		0.194276	NS
**GII Normal**		0.6930648		0.672383	NS
**Mean Gained Chromosomes**		2.37		1.67	NS
**Mean Lost Chromosomes**		1.26		2.09	NS

^1^ Statistical analysis was carried out using Student’s t test. ^2^ It has been carried out on the maximum number of patients with the specimen available. EOCRC: Early-onset colorectal cancer; EORC: Early-onset rectal cancer: LOCRC: Late-onset colorectal cancer; LORC: Late-onset rectal cancer; NS: Not significant; SD: Standard deviation; UICC: International Union Against Cancer; S- and/or MCRC: synchronous and/or metachronous; LS: Lynch syndrome; MMR: Mismatch repair; MSI: Microsatellite Instability; CIMP: CpG island methylator phenotype; MSS: Microsatellite stable; GII: Genomic instability index.

**Table 4:** Summary of the main specific differential chromosomal regions between each age-of-onset group, for each colon location.

	Chromosomal Segment	RIGHT-SIDED		LEFT-SIDED COLON		RECTUM		
		EO (*n* = 13)	LO (*n* = 35)	EO (*n* = 27)	LO (*n* = 19)	EO (*n* =2 0)	LO (*n* = 32)	
		**%**	**%**	**%**	**%**	**%**	**%**	
chr1	p32.3-22.2	0	**51**					
chr1	p21.3-11.2	0	**51**					
chr2	p25.2	0	**51**					**RIGHT COLON**
chr2	p11.2	**54**	20					
chr10	q11.21-11.22	**70**	23					
chr14	q11.1-11.2	**85**	37					
chr16	p13.12-13.11	**54**	11					
chr17	p11.2	**62**	29					
chr18	p11.32-11.21	0	**54**					
chr18	q21.1-21.1	8	**51**					
chr18	q21.31-21.33	8	**54**					
chr7	q11.22-21.11	15	**63**					
chr7	q22.1-31.33	15	**57**					
chr7	p12.2-11.2	15	**54**					
chr7	q11.21	15	**54**					
chr9	q12-13	0	**51**					
chr9	q33.3	**62**	29					
chr9	q34.12-34.13	**62**	29					
chr13	q11	8	**51**					
chr21	q22.3	**62**	26					
								
chr1	p12-q21.1			**63**	10			**LEFT COLON**
chr5	q13.1-13.2			**67**	16			
chr9	p12-q24.22			**59**	21			
chr9	q31.3-33.1			**52**	16			
chr11	p11.12-q12.1			**63**	21			
chr11	q14.1-14.3			**59**	21			
chr15	p11.1-q11.2			0	**26**			
chr19	p13.12-12			**59**	26			
								
chr1	p36.32-36.13					5	**41**	**RECTUM**
chr1	p36.23-36.22					**37**	12	
chr3	p21.31-21.1					**42**	32	
chr5	q13.2					5	**53**	

EO: Early-onset; LO: Late-onset; Chr: chromosome; Green: gained regions; Red: lost regions. Percentages shown in bold indicate frequencies that are at least twice as high in one age-of-onset group as in the other.

## Supplementary Materials

Supplementary materials can be found at https://www.mdpi.com/1422-0067/20/4/968/s1. Table S1: Differential chromosomal regions between each age-of-onset group, for each colon location.

## Abbreviations

aCGH	array of Comparative Genomic Hybridization
CIMP	CpG Island Methylator Phenotype
CIN	Chromosomal Instability
CRC	Colorectal cancer
CMS	Consensus Molecular subtype
CNA	Copy Number Alterations
DFS	Disease-Free survival
EOCC	Early-onset colon cancer
EOCRC	Early-onset colorectal cancer
EORC	Early-onset rectal cancer
GEO	Gene Expression Omnibus
LOCC	Late-onset colon cancer
LOCRC	Late-onset colorectal cancer
LORC	Late-onset rectal cancer
LS	Lynch syndrome
MCRC	Metachronous colorectal cancer
MMR	Mismatch Repair
MSI	Microsatellite instability
MSS	Microsatellite stability
OS	Overall survival
SCNA	somatic copy number alterations
SCRC	Synchronous colorectal cancer
SD	Standard Deviation
