# Supplementary material for: Clinical and Molecular Comparative Study of Colorectal Cancer Based on Age-of-Onset and Tumor Location: Two Main Criteria for Subclassifying Colorectal Cancer
Source: Int J Mol Sci. 2019 Feb 22;20(4):968. doi: 10.3390/ijms20040968 (PMC6413061; doi:10.3390/ijms20040968)
Supplement: Supplementary file 1 [file ijms-20-00968-s001.pdf]

Table S1. Differential chromosomal regions between each age-of-onset group, for each colon location.

		RIGHT CC		LEFT CC		RECTAL CANCER	
		Early-onset	Late-onset	Early-onset	Late-onset	Early-onset	Late-onset
		%	%	%	%	%	%
chr1	p36.32	0	**37**				
	p36.32-36.13					5	**41**
	p36.32-36.31	**46**	14	**29**	0		
	p36.31	0	**34**				
	p36.23-36.22					**37**	12
	p36.13-36.12					5	**32**
	p35.3-35.2	**46**	14				
	p35.2-34.3	0	**31**			**0**	**32**
	p34.3	**46**	14				
	p32.3-22.2	0	**51**				
	p21.3-11.2	0	**51**				
	p12-q21.1			**63**	10		
	q21.1-21.2	0	**40**				
	q21.1-21.2	**69**	23	**55**	10		
	q22-23.1					0	**21**
	q23.2-24.3	0	**29**				
	q25.1-31.3	12	**34**				
	q32.1-32.2	12	**34**				
	q32.2	0	**37**				
	q32.3	0	**40**				
	q41	0	**34**				
	q42.2	0	**31**				
	q42.2	0	**34**				
	q42.3	0	**37**				
	q43	0	**31**				
chr2	p25.2	0	**51**				
	p25.1-24.1	0	**43**	**41**	5		
	p23.2-22.2	0	**37**				
	p22.1-21	0	**37**				
	p16.3-16.2	0	**43**				
	p16.1	0	**46**				
	p14-13.3	0	**40**				
	p12	0	**43**				
	p11.2	**54**	20				
	p11.2-11.1			**48**	10		
	p11.2	0	**37**				
	p11.1-q11.2	0	**29**				
	q11.2-12.1	0	**43**				
	q12.2-12.3	0	**43**				
	q12.3-21.1	0	**37**				
	q13			**48**	16		
	q14.1-14.2	0	**43**	**37**	5		
	q14.3	0	**49**	**33**	0		
	q21.1	0	**37**	**26**	0		
	q21.2	0	**37**	**26**	0		
	q22.1-35	0	**40**	**22**	0		
	q32.3					5	**32**
	q35-36.1					5	**32**

	q36.1	0	**31**				
	q36.3	0	**34**				
	q37.1-37.3	0	**34**				
chr3	p25.3	0	**31**				
	p21.31-21.1					**42**	32
	q26.31			**22**	0		
chr4	q31.3			**33**	5		
	q32.1			**37**	5		
chr5	q13.1-13.2			**67**	16		
	q13.2					5	**53**
	p13.3	0	**29**				
chr6	p12.2-12.2			**33**	5		
	p11.2-11.1			**44**	11		
	q25.3	0	**29**				
chr7	p15.2	8	**40**				
	p14.3-14.1	8	**40**				
	p13-12.3	8	**46**				
	p12.2-11.2	15	**54**				
	q11.21	15	**54**				
	q11.22-21.11	15	**63**				
	q21.11-21.12	8	**40**				
	q21.13-21.1	8	**51**				
	q21.3	8	**40**				
	q22.1-31.33	15	**57**				
	q32.3-34	8	**46**				
	q35	8	**46**				
chr8	p23.1-21.3	0	**31**				
	q22.1	8	**40**				
	q24.21	8	**43**				
	q24.21-24.22			**44**	11		
chr9	p13.3-13.2					0	**20**
	p13.1	8	**46**				
	p12-11.2	0	**46**				
	p12-q24.22			**59**	21		
	q12-13	0	**51**				
	q21.11	0	**49**				
	q21.11-21.12					0	**26**
	q21.31-21.32					0	**26**
	q31.3-33.1			**52**	16		
	q33.2			**22**	0		
	q33.3	**62**	29				
	q34.12-34.13	**62**	29				
chr10	p15.3	0	**34**				
	p15.3-15.1					**21**	3
	p14	0	**31**				
	p14					**21**	3
	p12.31					0	**21**
	q11.21-11.22	0	**40**			8	**23**
	q11.21-11.22	**70**	23				
	q22.2-23.32	0	**40**	**48**	16		
	q23.32-23.33					**21**	3
	q23.33-24.1					0	**21**
	q26.12-26.13	0	**29**				

	q26.13-26.2	0	**29**				
chr11	p15.5-15.4			**37**	5		
	p15.2			**48**	16		
	p11.12-q12.1			**63**	21		
	q14.1-14.3			**59**	21		
	q24.13-24.3	0	**37**				
chr12	p13.31	8	**40**				
	p13.2-13.1	0	**31**				
	p12.3	0	**29**				
	p12.2	0	**29**				
	p12.1	0	**29**				
	p11.21	0	**37**				
	q24.13-24.32	0	**31**				
	q24.32-24.33	0	**31**				
chr13	q11	8	**51**				
	q12.11-12.13	8	**46**				
	q12.2-12.3	8	**40**				
	q14.11-14.13	8	**43**				
	q14.2-14.3	8	**40**				
	q31.3-32.3	8	**49**				
chr14	q11.1-11.2	**85**	37				
	q11.22			**44**	5		
	q12	8	**40**				
chr15	p11.1-q11.2			0	**26**		
	q11.2	8	**40**				
	q14	8	**46**				
	q15.3	8	**43**				
	q21.1	8	**46**				
	q21.2-26.3	0	**37**				
chr16	p13.3	0	**29**				
	p13.12-13.11	**54**	11				
	p12.2-p11.2	**31**	6				
	q12.1-q22.1	0	**31**				
	q21					**21**	3
	q23.1-q23.3	0	**29**				
chr17	p13.3			**44**	11		
	p13.3					0	**24**
	p13.1-12			**30**	0		
	p11.2	**62**	29				
	q22-23.1			**48**	16		
	q24.2-24.3	0	**29**				
chr18	p11.32-11.21	0	**54**				
	p11.31-11.22					**21**	3
	q11.2-12.3	8	**49**				
	q21.1-21.2	8	**51**				
	q21.31-21.33	8	**54**				
	q22.1-23	8	**46**				
chr19	p13.12-12	0	**31**	**59**	26		
	q13.12			**48**	16		
chr20	p12.2-11.23	0	**29**				
chr21	q22.3	**62**	26				
	q22.3	0	**29**				
chr22	q13.31-13.32	0	**29**				

CC: Colon Cancer; Chr: chromosome. Green: gained regions. Red: lost regions. Percentages shown in bold indicate frequencies that are at least twice as high in one age-of-onset group as in the other.
